# Meta AI literacy scale: Further validation and development of a short version

**DOI:** 10.1016/j.heliyon.2024.e39686

**Published:** 2024-10-22

**Authors:** Martin J. Koch, Astrid Carolus, Carolin Wienrich, Marc E. Latoschik

**Affiliations:** a Media Psychology, Julius-Maximilians-University Würzburg, Oswald-Külpe-Weg 82, 97074, Würzburg, Germany; b Psychology of Intelligent Interactive Systems, Julius-Maximilians-University Würzburg, Emil-Fischer-Straße 50, 97074, Würzburg, Germany; c Human-Computer-Interaction, Julius-Maximilians-University Würzburg, Emil-Fischer-Straße 50, 97074, Würzburg, Germany

## Abstract

The concept of AI literacy, its promotion, and measurement are important topics as they prepare society for the steadily advancing spread of AI technology. The first purpose of the current study is to advance the measurement of AI literacy by collecting evidence regarding the validity of the Meta AI Literacy Scale (MAILS) by Carolus and colleagues published in 2023: a self-assessment instrument for AI literacy and additional psychological competencies conducive for the use of AI. For this purpose, we first formulated the intended measurement purposes of the MAILS. In a second step, we derived empirically testable axioms and subaxioms from the purposes. We tested them in several already published and newly collected data sets. The results are presented in the form of three different empirical studies. We found overall evidence for the validity of the MAILS with some unexpected findings that require further research. We discuss the results for each study individually and also together. Also, avenues for future research are discussed. The study's second purpose is to develop a short version (10 items) of the original instrument (34 items). It was possible to find a selection of ten items that represent the factors of the MAILS and show a good model fit when tested with confirmatory factor analysis. Further research will be needed to validate the short scale. This paper advances the knowledge about the validity and provides a short measure for AI literacy. However, more research will be necessary to further our understanding of the relationships between AI literacy and other constructs.

## Highlights

• The paper collects evidence concerning the validation of the Meta AI Literacy Scale.
• A short version (10 items) of the original Meta AI Literacy Scale is developed.
• Four new and published data sets are combined.

## 1 Introduction

Artificial Intelligence literacy (short: AIL or AI literacy) is one of the current “hot topics” in both academia and the public. AIL is “a set of competencies that enables individuals to critically evaluate AI technologies, communicate and collaborate effectively with AI; and use AI as a tool online, at home, and in the workplace” [1]. An increasing number of publications are dedicated to the conceptualization of A I literacy [[1], [2], [3]], the improvement of AI literacy in different target groups [[4], [5], [6], [7], [8], [9]], and the measurement of AI literacy using self-assessment scales [[10], [11], [12], [13], [14], [15]] or tests [16]. The majority of the recently published instruments in this context rely on self-report questionnaires (i.e. the self-assessment of one's own AI literacy instead of performance-based measures) raising the questions of their criteria of quality (e.g. objectivity and reliability of the results). Questionable reliability and validity of a measurement results in poor estimates of the construct of interest, which makes testing of hypotheses and informed decisions impossible. Researchers need valid measurements to answer their research questions and practitioners need valid instruments to address their requirements, e.g., select personnel, identify knowledge in teams, or derive personnel development plans. Recently published measures of AI literacy [[10], [11], [12], [13], [14], [15]] have mostly only been tested regarding their factorial structure so far, which points out the need for further measures to collect evidence for the validity of the instruments. Furthermore, the published instruments include a large number of self-report items (e.g. MAILS: 34 items; MAIRS-MS by Ref. [11]: 22 items; AIL Scale by Ref. [14]: 16 items) making them viable for research purposes but limiting their practicality and applicability for everyday use.

The present paper addresses the need for validation and the need for less time-consuming data collection for the Meta AI Literacy Scale by Carolus, Koch et al. [10]. Thus, this study aims for both contributions to the validation of the long scale and the development of a short version of the instrument.

## 2 Theoretical background

### 2.1 The Meta AI literacy scale

The Meta AI Literacy Scale (MAILS; [10]) is a scale for measuring AI literacy and other psychological competencies (problem-solving, learning, self-efficacy, persuasion literacy) that are assumed to support successful interaction with AI [[17], [18], [19]]. The design of the instrument was originally based on the AI literacy conceptualization by Ng et al. [3] concerning AI literacy (not concerning the supplementary psychological competencies) and the taxonomy of Bloom [20]. Ng and colleagues [3] differentiate between the following domains of AI literacy: Knowing and understanding AI, using and applying AI, evaluating and creating AI, and making human-centered considerations about AI ethics. However, empirical data in an initial factor-analytical review indicate a slightly different structure of the items as described in the following paragraph [10].

The MAILS includes 34 items, 18 items focusing on *AI literacy*, four focusing on the ability to *create AI*, and 6 each focusing on *AI self-efficacy* and *AI self-competency* when dealing with AI. The AIL conceptualization has been derived from extensive literature analyses [3] and was modified after the collection of empirical data.

• Six items measure the ability to use and apply AI (e.g. I can operate AI applications.“, „I can communicate gainfully with artificial intelligence.“).
• Another six items measure knowledge and understanding of AI (e.g. „I know definitions of artificial intelligence.“, „I can assess what advantages and disadvantages the use of an artificial intelligence entails.“).
• Unlike the conceptualization by Ng et al. [3], this scale also includes items on the evaluation of AI and future possible uses of AI.
• Also contrary to their concept, the ability to detect AI (as described by Refs. [1,15]) is a separate sub-factor of AIL measured with three items (e.g. „I can tell if I am dealing with an application based on artificial intelligence.“).
• Three items measure the ability to make ethical considerations regarding AI (e.g. „I can weigh the consequences of using AI for society.“).

The ability to create AI was measured using four items (e.g. „I can program new applications in the field of “artificial intelligence”.”). In contrast to the original concept of Ng et al. [3], structural equation modelling revealed that the ability to create AI was not a subdimension of AI literacy but only a correlate.

Further twelve items measure the additional psychological competencies. The factor AI self-efficacy (as described by [17], [18], [19]) includes self-efficacy in problem-solving ability as desribed by Ajzen [21] related to AI (e.g. „I can rely on my skills in difficult situations when using AI.“) and learning (e.g. „Despite the rapid changes in the field of artificial intelligence, I can always keep up to date.“) measured with three items each. The latent factor of AI self-competency measures the ability to manage one's information (e.g. „I don't let AI influence me in my decisions.“) and emotions (e.g. „I keep control over feelings like frustration and anxiety while doing things with AI.“) with three items each. To summarize, unlike other available measures of AI literacy, the MAILS includes the ability to create AI as a related construct and also measures psychological competencies that are expected to be helpful or needed for the prolonged use of AI.

Participants answer the MAILS using an 11-point Likert scale from 0 to 10 with descriptions for the extrema („A value of 0 means that an ability is not at all or hardly pronounced.” And „A value of 10 means that an ability is very well or almost perfectly pronounced.”). Following Bandura [22], this scale can be understood as an individual's “strength of their belief in their ability to execute the requisite activities“ (p. 312). Moreover, it can be considered metric compared to other types of scales with additional descriptions or fewer points [23].

A highly up-to-date overview article on AI literacy self-assessment scales and tests was recently published [24]. The validity of the self-report measurement instruments for AI literacy [11,12,14,15] has only been tested regarding their factorial structure. However, to measure AI Literacy solidly in both practical and scientific projects, it is important to advance the validation of the measurement. Although the validity of the test scores can never be conclusively proven, it is important to collect and examine evidence for the validity of the intended test score interpretation [25]. Furthermore, again for both practice and research, a methodologically sound short version of the MAILS would be beneficial. The MAILS consists of 34 items, which limits its economic use.

### 2.2 Empirical testing of a nomological network for the validation of the Meta AI literacy scale

The concept of validity underwent many changes during its history [26]. Since the 1950s, more and more different types of validity have been distinguished (e.g. criterion validity, predictive validity, content, validity, face validity, convergent, and discriminant validity; [25]). Today, validity is considered a characteristic of a specific interpretation of a measurement instrument not a characteristic of the measurement instrument itself. Current scientific discourse refers to theory-based axioms about the construct to be measured and its relationships to other constructs. These axioms are integrated into a nomological network which can be empirically tested (Table 1). If the confirmation of the axioms fails, the axioms will need to be rejected, or the measurement procedure will be revised.

**Table 1:** Overview of the studies testing the axioms. Please refer to section 2.2 for further explanations on the axioms.

study		I	II	III
original publication of the data		Carolus et al., 2023	Koch et al., sub.	
Tested axioms	1a	*CFA: factorial structure*	*CFA: factorial structure*	
	1b		correlate: other AIL scales	
	1c	*correlations: attitude, OT*	correlations: attitude	
	1d			correlations: AIL test
	1e			
	2a	correlations: use of AI		correlations: use of AI
	2b			correlations: learning of AI
	2c			

Note. Analyses printed in italics have been published before. CFA = Confirmatory Factorial Analysis; OT = Openness to technology.

To validate a measurement instrument, the first step is to the purpose for which the test scores are to be interpreted. The second step is to derive empirically testable axioms. The third step is to collect empirical evidence from existing or newly collected data to test the axioms [25].

Following this procedure, in step 1 of the validation procedure, we formulated two intended uses for the MAILS. First, the scale should be able to explain individual AI literacy and its related psychological competencies. The second goal of the MAILS is the evaluation of interindividual differences in AI literacy and psychological competencies. Step 2 of the validation procedure is to derive empirically testable axioms. Thus axiom 1 postulates that the MAILS scores are indicators of the individual level of AI literacy and its associated psychological competencies. From axiom 1 empirically supported sub-axioms are derived. These sub-axioms further specify the axioms in relation to the intended uses of the tests, explicate how they can be empirically tested, and are supported by literature.

• Axiom 1a assumes that the answers to the scale (manifest variables) can be traced back to the different ability factors of AI literacy and supplementary psychological competencies (latent variables). Following the conceptualization by Ng et al. [3], we also include further competencies and latent factors and the original publication of the scale (see section 2.1; [10]).
• Axiom 1b postulates that the answers to the MAILS are highly correlated with the results of other AI literacy scales as they should measure the same construct (called convergent validity, [27]).
• Axiom 1c postulates that the answers to the MAILS show medium correlations with related constructs (attitude towards AI, openness towards technology). As these constructs are relevant for the use of AI, people who have higher levels (e.g. a higher positive and lower negative attitude as well as a higher general openness towards technology) interact more frequently with AI and thus become more proficient in its use (i.e. higher AI literacy). Research from other areas shows that attitude is positively related to behavior [[28], [29]]. This might also include openness which can be considered a positive attitude towards new technology and reflects the willingness to use new technology [30] – like AI.
• Axiom 1d postulates that the answers on the scale are highly correlated with AI-related knowledge (knowledge about AI, about interacting with AI). Like axiom 1b, axiom 1d postulates that instruments that measure the same construct should be correlated [27].
• Axiom 1e postulates that AIL is not highly correlated with more general cognitive abilities (IT literacy, data literacy, intelligence; divergent validity, [27]).

The second goal of the MAILS is the evaluation of interindividual differences in AI literacy and psychological competencies. In this context, as described in step 2 of the validation process, three distinct axioms were derived.

• Axiom 2a postulates that the frequent prior use of AI comes with higher MAILS values.
• Further, axiom 2b postulates that the use of formal and informal learning opportunities regarding AI leads to higher MAILS values.
• Lastly, axiom 2c postulates that individuals who take advantage of a formal or informal learning opportunity have a higher value after learning than they did before learning.

#### 2.2.1 Preliminary results

So far, two empirical studies included information on step 3 of the validation process, the collection of empirical data concerning the axioms. They tested the first axiom by examining the factorial structure in a German-speaking and an English-speaking sample (axiom 1a) using a confirmatory factor analysis. In the first study by Carolus, Koch, and colleagues [10], a factorial structure for the MAILS was confirmed. The χ2-test became significant (*N* = 300; χ2(513) = 886.87, *p* < .001). However, the other model fit indices showed a good model fit (CFI = .93, RMSEA = .06, 95 %-CI [.05, .06], SRMR = .08). CFI >.9 and RMSEA <.08 indicate an acceptable model fit, while an SRMR >.08 indicates no good fit [31]. The second sample (*N* = 219) also confirmed the measurement model of the MAILS as it showed a good model fit (χ2(513) = 781.49, *p* < .001, CFI = .95, RMSEA = .05, 95 %-CI [.05, .06], SRMR = .06). Moreover, positive correlations of the MAILS with related constructs as postulated in axiom 1 c in German- and English-speaking samples have been shown [10]: AI literacy, AI self-competency, and AI self-efficacy correlated significantly positive with most of the factors of openness to technology (except AI self-efficacy and competence when dealing with new technology) and positive attitude towards AI. Interestingly, the ability to create AI was not correlated with control over new technology and negatively correlated with competence when dealing with new technologies. Further, the ability to create AI and AI self-efficacy were not correlated with negative attitudes toward AI.

### 2.3 Research questions and overview of studies

The paper aims for (1) a deeper understanding of the validity of the Meta AI Literacy Scale and (2) a short version of the scale. For the first purpose, we combined evidence from three data sets from German- and English-speaking samples. Table 1 provides an overview of the axioms and the three studies. For studies I and II, data is used that has been published before [10] or will be published (Koch et al., submitted). Study II is based on unpublished data. For all studies, R (R Core Team, 2016) was used to analyze the data. Axiom 1a was tested and published in the original publication on the scale [10] and is shortly described in section 2.2.1. We were not able to generate empirical results concerning axioms 1e and 2c. They, thus, remain untested so far.

## 3 Methods and materials

### 3.1 Study I: Test of axiom 2a (correlation with prior use of AI)

The goal of the first study was to test if interindividual differences in the prior use of AI affect the scores of the Meta AI Literacy scale (axiom 2a). The axiom postulates that more prior use of AI to higher values regarding AI literacy as reported with the MAILS. The data for this study was already published (Table 1).

Data were collected online on the 3rd and November 4, 2022. The survey tool SoSci-Survey [32] was used to collect data and participants were recruited using Prolific. co [33]. Before participation, participants gave informed consent. The average completion time was 16.08 min (SD = 5.77) resulting in compensation worth ₤3.00.

Ethical approval of this study was not requested following the suggestions of the German Research Foundation (DFG, (https://www.dfg.de/de/foerderung/antrag-foerderprozess/faq/geistes-sozialwissenschaften). For studies of all social sciences including research with human participants, ethical approval is only required when using identifiable data, when including patients or other vulnearbale groups, when using material which ilicits strong emotion (e.g. stress or traumatic experiences) or physical danger or pain, when the participants are not informed about the aim of the study or are deliberately misled as experimental manipulation, or are exposed in any other way to special social, legal, financial, or professional risks. In addition, for psychological studies, ethical approval is required when using electric or magnetic stimulation or psychopharmacological examinations. Risks and harm to the participants are not to be expected in the simple surveys on non-sensitive topics we conducted.

#### 3.1.1 Sample

The sample consisted of 300 German-speaking individuals. The average age was 32.13 years (SD = 11.66 years, ranging from 18 to 72 years). Most participants lived in Germany (76.51 %) or Austria (7.12 %). Other participants lived in the United Kingdom (4.98 %), Switzerland (2.85 %), Netherlands (2.14 %), Spain (1.78 %), France (1.07 %), Poland, Portugal, the United States (each .71 %), Australia, Greece, Italy, or South Africa (each .36 %). 145 participants considered themselves female (48.33 %), 152 participants identified as male (50.67 %), and 3 participants identified as diverse (1.00 %). The majority either worked full-time (30.00 %) or part-time jobs (23.67 %), or were not engaged in paid work (24.00 %). No information on employment status was available for 22.33 % of the participants.

#### 3.1.2 Measures

*Prior use of AI:* Participants indicated their *prior use of AI* by rating three statements (“I use artificial intelligence at work”, “I use artificial intelligence at school/university”, and “I use artificial intelligence in my everyday life”) on an 11-point Likert scale (0 = “never or only very rarely” to 10 = “very often”).

*AI Literacy:* The *Meta AI Literacy Scale* as described in section 2.1 in a German version was used to measure AI literacy. Please note that in this study, additional items originally designed for the MAILS were included for the data collection because we developed more items than those included in the final instrument. These items were dropped during the scale refinement. Also, the items were not presented in the order described by Carolus et al. [10]. The MAILS consists of 34 items. The scales and subscales of the MAILS were described in section 2.1 of this article. Each item is scored on a scale from 0 to 10. There is no scale labelling to achieve an approximate metric scale level. As additional information, the scale is described above: a value of 0 means that the ability is hardly or not at all pronounced, whereas a value of 10 means that the ability is very well or almost perfectly pronounced. For each of the scales, a sum score was calculated. Internal consistencies (Cronbach's α) for the total AI literacy and the subscales (Use AI, Create AI, and AI Self-efficacy) were high (α > .90). Internal consistencies for Know & understand AI, Learning, and problem-solving were good (α > .90). The internal consistencies for Detect AI, AI Self-and emotion regulation still acceptable (α > .70). Only the internal consistency of persuasion literacy was questionnable (α = .66). We nonetheless decided to keep the scale as in the original for the purpose of the validation.

#### 3.1.3 Results

The average scores (and standard deviation) were *M* = 1.84 (*SD* = 2.79) for the use of AI at work, *M* = 1.21 (*SD* = 2.31) for the use of AI at school/university, and *M* = 3.73 (*SD* = 3.03) for everyday life respectively. Each item was answer by all participants (*N* = 300). The sample showed a rather low use of AI. To test the axiom 2a, we calculated correlations of each of the MAILS scales' mean scores and subscales mean scores and the three values for the prior use of AI at work, at school/university, and in everyday life (Table 2).

**Table 2:** Pearson correlations of the use of AI at work, school/university, and in everyday life with the scales and subscales of the MAILS.

	Use of AI		
	at work	at school/university	in everyday life
AIL	.35∗∗∗	.29∗∗∗	.34∗∗∗
Create AI	.36∗∗∗	.36∗∗∗	.13∗
AISC	.20∗∗∗	.12∗	.30∗∗∗
AISE	.34∗∗∗	.27∗∗∗	.34∗∗∗
Know & understand AI	.35∗∗∗	.29∗∗∗	.34∗∗∗
Use AI	.37∗∗∗	.24∗∗∗	.46∗∗∗
AI Ethics	.27∗∗∗	.23∗∗∗	.25∗∗∗
Detect AI	.28∗∗∗	.21∗∗∗	.29∗∗∗
Emotion regulation	.17∗∗	.09	.31∗∗∗
Persuasion literacy	.18∗∗	.13∗	.21∗∗∗
Problem-solving	.31∗∗∗	.25∗∗∗	.32∗∗∗
Learning	.33∗∗∗	.26∗∗∗	.32∗∗∗

Note. ∗*p* < .05, ∗∗*p* < .01, ∗∗∗*p* < .001; AIL = AI literacy, AISC = AI self-competency, AISE = AI self-efficacy.

Results revealed that the prior use of AI at work, in everyday life, and at school/university were significantly correlated to all scales and subscales of the MAILS with one exception. Prior use of AI at school/university was not correlated the self-assessed ability to regulate one's own emotions when interacting with AI. All significant correlations were of small to moderate effect size.

#### 3.1.4 Discussion

Confirming axiom 2a, we found positive significant correlations between the prior use of AI at work, at school/university, and in everyday life with all scales and subscales of the MAILS (axiom 2a). These findings support the assumption of the axiom that more frequent use of AI is associated with higher MAILS scores indicating higher AI literacy. Thus, the MAILS can detect the differences between individuals who use AI more often compared to those who use it less frequently. Consequently, this aspect of validity can therefore be regarded as given. Some correlations with use of AI at school/university are non-significant and most are descriptively smaller than the correlations with the other two items. One possible reason is a potentially lower validity of the item, as many participants no longer went to school or university (we, unfortunately, have no information on this) and, thus, were not able to correctly answer the item. Similarly, we cannot be sure how many participants have the opportunity to engage with AI in the work environment (only 53.67 % of participants reported working either full or part time). Furthermore, and we have no reliable way of knowing how they interpreted what it means to “use AI” (German: “AI benutzen”). The word “use” and its German eqivalent are problematic, because of the vagueness. “Using” AI might mean that they are AI developers, taking an AI course, or are interacting with some AI system. This issues can reduce the accuracy of our findings.

### 3.2 Study II: Test of axiom 1b (correlation with attitude towards AI) and 1c (correlation with other AIL scales)

The first goal of the study was to test if correlations between AI Literacy and positive and negative attitudes towards AI can be identified using the MAILS (axiom 1b). We hypothesized that (a) individuals with higher positive attitudes towards AI show higher AI literacy and (b) individuals with lower negative attitudes towards AI show higher AI literacy. Moreover, study II aims to test axiom 1c postulating that the MAILS is correlated with other self-report instruments that are also intended to measure AI literacy.

We collected the data online from 149 individuals from February 9th to 13th 2023. As in Study I, we used the survey tool SoSci-Survey [32] and the recruitment tool Prolific. co [33]. The average completion time was 17.45 min (SD = 15.35). Before participation, all participants gave informed consent. After we reviewed the quality of the data, the participants received compensation worth ₤3.00 for their participation.

Ethical approval of this study was not requested as suggested by the German Research Foundation (DFG) and as described in detail in section 3.1.

#### 3.2.1 Sample

The sample consisted of 149 English-speaking individuals from the United Kingdom (*n* = 148) and the United States (n = 1). The average age was 39.81 years (*SD* = 13.21), ranging from 18 to 79 years. Of all participants, 56.37 % identified as female, 42.95 % identified as male, and .67 % as diverse. 9.13 % of the participants are students. One fifth was not engaged in paid work (19.64 %), while others were distributed across full-time (34.69 %) and part-time (19.05 %) employment categories. For 26.53 %, no information on employment status was available.

#### 3.2.2 Measures

*Attitude towards AI:* This construct was measured using the *General Attitudes Towards Artificial Intelligence Scale* [34]. It measures positive (e.g., “For routine transactions, I would rather interact with an artificially intelligent system than with a human.“) and negative attitudes towards artificial intelligence (e.g., “I think artificially intelligent systems make many errors.“). Participants were asked to rate their agreement with the 20 statements using a 5-point Likert scale (“Do not agree at all”, “Do not agree”, “Neutral”, “Somewhat agree”, and “Fully agree”). A mean score was calculated for positive and negative attitudes each. Internal consistencies for both scales were high for positive attitude (α = .91) and acceptable for negative attitude (α = .79).

*AI Literacy:* Again, we used the *Meta AI Literacy Scale* as described in section 2.1 to measure AI literacy. However, this time we used the English items [10]. We used the instructions and items as described but mixed them with items from other AI literacy measurement instruments and presented them in a randomized order. The internal consistencies of all scales and subscales of the MAILS were good (α > .80) or high (α > .90) except for persuasion literacy which still was acceptable (α = .74). We calculated sum scores for each of the scales.

Additionally, AI literacy measures included the AI Literacy Scale [15], the Medical AI Readiness Scale for Medical Students (MAIRS-MS) [11], and the AI Literacy Scale [14]. The instruments were added as they were among the most cited AI literacy self-report instruments and freely available in English. The scale by Wang et al. [15] measures AI literacy across the factors of Awareness (e.g., „I can distinguish between smart devices and non-smart devices”; α = .50), Usage (e.g., „I can skilfully use AI applications or products to help me with my daily work”; α = .63), Evaluation (e.g., „I can evaluate the capabilities and limitations of an AI application or product after using it for a while”; α = .80), and Ethics (e.g., „I always comply with ethical principles when using AI applications or products”; α = .35) using three items per factor. Despite the low internal consistencies for some of the scales, we decided to keep them in their original form.

The MAIRS-MS [11] consists of 22 items grouped in the factors Cognition (e.g., „I can define the basic concepts of data science”; α = .92), Ability (e.g., „I can use AI-based information in combination with my professional knowledge”; α = .94), Vision (e.g., „I can explain the limitations of AI technology”; α = .86), and Ethics (e.g., „I can use data in accordance with legal and ethical norms”; α = .73). We removed all references to the medical context from the items. The internal concistencies were good (α > .80) or high (α > .90) except for Ethics which still was acceptable (α = .73).

The last additional AI literacy scale [14] consists of one general AI literacy scale and four subscales. General AI literacy is measured using three items (e.g., „Considering all my experience, I am relatively proficient in the field of AI”; α = .87). The subscales were measured using three items each for AI technology knowledge (e.g., „I have knowledge of the types of technology that AI is built on”; α = .87), Human actors in AI knowledge (e.g., „I have knowledge of which human actors beyond programmers are involved to enable human-AI collaboration”; α = .82), and AI steps knowledge (e.g., „I have knowledge of the input data requirements for AI”; α = .88). Two items per scale were used for AI usage experience (e.g., „I have experience in interaction with different types of AI, like chatbots, visual recognition agents, etc”; α = .74) and AI design experience (e.g., „I have experience in designing AI models, for example, a neural network”; α = .78). All internal consistencies were good or acceptable. For each scale, a sum score was calculated.

#### 3.2.3 Results

Pearson regressions between all scales of the MAILS and the positive and negative attitudes towards AI and the additional AI literacy measures revealed that all scales except persuasion literacy were positively correlated with a positive attitude toward AI (Table 3). The correlations were of small to big effect size. Unlike expected, only AI self-efficacy, Use AI, emotion regulation, and problem-solving were negatively correlated to a negative attitude toward AI. These effects were small (|.1| < *r* < |.3|).

**Table 3:** Pearson correlations between all scales of the MAILS and the positive and negative attitude towards AI and the additional AI literacy measures. The additional scales are presented in the following order separated by vertical lines: Wang et al. [15], Karaca et al. [11], and Pinski & Benlian [14].

	AIL	Create	AISC	AISE	Use	Know	Ethics	Detect	ER	PL	PS	Learn
Positive att	.55∗∗∗	.26∗∗	.35∗∗∗	.48∗∗∗	.56∗∗∗	.55∗∗∗	.45∗∗∗	.27∗∗∗	.44∗∗∗	.15	.42∗∗∗	.48∗∗∗
Negative att	−.13	−.06	−.14	−.17∗	−.17∗	−.11	−.08	−.05	−.18∗	−.06	−.17∗	−.14

Awareness	.52∗∗∗	.29∗∗∗	.56∗∗∗	.49∗∗∗	.44∗∗∗	.44∗∗∗	.48∗∗∗	.59∗∗∗	.45∗∗∗	.54∗∗∗	.48∗∗∗	.44∗∗∗
Usage	.82∗∗∗	.44∗∗∗	.57∗∗∗	.78∗∗∗	.83∗∗∗	.74∗∗∗	.72∗∗∗	.54∗∗∗	.59∗∗∗	.40∗∗∗	.74∗∗∗	.73∗∗∗
Evaluation	.85∗∗∗	.52∗∗∗	.66∗∗∗	.85∗∗∗	.81∗∗∗	.78∗∗∗	.80∗∗∗	.59∗∗∗	.64∗∗∗	.50∗∗∗	.82∗∗∗	.78∗∗∗
Ethics	.71∗∗∗	.38∗∗∗	.64∗∗∗	.65∗∗∗	.64∗∗∗	.66∗∗∗	.67∗∗∗	.56∗∗∗	.62∗∗∗	.49∗∗∗	.65∗∗∗	.59∗∗∗

Cognition	.85∗∗∗	.68∗∗∗	.60∗∗∗	.84∗∗∗	.76∗∗∗	.86∗∗∗	.77∗∗∗	.58∗∗∗	.58∗∗∗	.46∗∗∗	.81∗∗∗	.78∗∗∗
Ability	.93∗∗∗	.54∗∗∗	.67∗∗∗	.89∗∗∗	.91∗∗∗	.88∗∗∗	.78∗∗∗	.65∗∗∗	.65∗∗∗	.49∗∗∗	.86∗∗∗	.83∗∗∗
Vision	.84∗∗∗	.45∗∗∗	.64∗∗∗	.70∗∗∗	.71∗∗∗	.84∗∗∗	.81∗∗∗	.64∗∗∗	.60∗∗∗	.51∗∗∗	.67∗∗∗	.66∗∗∗
Ethics	.67∗∗∗	.29∗∗∗	.67∗∗∗	.58∗∗∗	.59∗∗∗	.60∗∗∗	.72∗∗∗	.53∗∗∗	.64∗∗∗	.51∗∗∗	.60∗∗∗	.51∗∗∗

Tech. know.	.78∗∗∗	.67∗∗∗	.55∗∗∗	.79∗∗∗	.69∗∗∗	.82∗∗∗	.70∗∗∗	.53∗∗∗	.51∗∗∗	.45∗∗∗	.73∗∗∗	.77∗∗∗
Human know.	.81∗∗∗	.63∗∗∗	.63∗∗∗	.75∗∗∗	.70∗∗∗	.81∗∗∗	.78∗∗∗	.59∗∗∗	.58∗∗∗	.52∗∗∗	.74∗∗∗	.69∗∗∗
Usage exp.	.78∗∗∗	.28∗∗∗	.61∗∗∗	.68∗∗∗	.80∗∗∗	.68∗∗∗	.61∗∗∗	.64∗∗∗	.60∗∗∗	.46∗∗∗	.65∗∗∗	.63∗∗∗
Design exp.	.42∗∗∗	.83∗∗∗	.26∗∗	.50∗∗∗	.37∗∗∗	.45∗∗∗	.37∗∗∗	.27∗∗∗	.25∗∗	.19∗	.48∗∗∗	.46∗∗∗
Steps know.	.72∗∗∗	.79∗∗∗	.46∗∗∗	.79∗∗∗	.65∗∗∗	.75∗∗∗	.66∗∗∗	.46∗∗∗	.45∗∗∗	.35∗∗∗	.73∗∗∗	.77∗∗∗
Total AIL	.85∗∗∗	.68∗∗∗	.55∗∗∗	.88∗∗∗	.81∗∗∗	.83∗∗∗	.74∗∗∗	.55∗∗∗	.55∗∗∗	.40∗∗∗	.82∗∗∗	.86∗∗∗

Note. ∗*p* < .05, ∗∗*p* < .01, ∗∗∗*p* < .001; AIL = AI literacy, Create = Create AI, AISC = AI self-competency, AISE = AI self-efficacy, Use = Use AI, Know = Know & understand AI, Ethics = AI Ethics, Detect = Detect AI, ER = Emotion regulation, PL = Persuasion literacy, PS = Problem solving, Learn = Learning. AIL refers to the scale of the MAILS while Total AIl refers to the scale of the AI Literacy Scale (Pinski & Benlian, 2023).

As postulated by the axiom 1c, the Meta AI Literacy Scales’ factorfactors showed small to high correlations with the subscales of all other AI literacy self-report instruments. The overall scale of AI Literacy was strongly correlated to the subscales of all instruments except Design experience with which it showed a medium correlation.

#### 3.2.4 Discussion

Confirming axiom 1b, the measures of the MAILS are significantly related to a positive attitude towards AI. Contributing to the validity of the instrument, the MAILS assesses AI literacy in such a way that its postulated association with a positive attitude towards AI literacy can be found. Unlike expected, negative attitude towards AI is not negatively related to all factorfactors of AI literacy as measured with the MAILS but only to a few select subscales. Possibly, negative attitude is not related to general AI literacy but only to specific subfactorfactors. Alternatively, the MAILS is not capable of capturing this specific aspect of AI literacy. Further, the validity is supported as the MAILS is positively correlated to other measures that presumably measure AI literacy (axiom 1c). This finding, however, is limited in so far as the validity of the other AI literacy self-report scales has not yet been tested besides the factorial structure. Ideally, validated measurement instruments are used to test this axiom.

### 3.3 Study III: Test of axioms 1d (correlations with an AI literacy test), 2a (correlation with prior use of AI), and 2b (correlation with learning about AI)

To test axiom 1d (correlation with an AI literacy test), the AI literacy test by Hornberger and colleagues [16] was used. This is the first performance-based AI literacy test that was developed independently of a specific intervention, validated, and peer reviewed. We hypothesized that individuals who rate their AI literacy to be high should also have a higher score in a knowledge test designed to measure AI literacy and vice versa. This should result in positive linear relationships between the MAILS's outcomes and the AI literacy test's results. However, as the test focuses on AI literacy knowledge, substantial correlations are only assumed for the subscale of Know & understand AI, the overall AI literacy scale, and the self-assessed ability to Create AI. Second, we aimed at testing axioms 2a and 2b hypothesizing that the outcomes of the MAILS are positively related to prior use of AI and prior learning about AI.

For this study, we collected the data online from 120 individuals on November 22nd, 2023, using SoSci-Survey [32] and Prolific. co [33], again. On average, it took 15.77 min (*SD* = 3.73) to complete the questionnaire. All participants gave informed consent before participation. After we reviewed the quality of the data, the participants received compensation worth ₤4.50 for their participation.

Ethical approval of this study was not requested, as risks and harm to the participants are not to be expected in simple surveys on non-sensitive topics. Basic ethical principles were adhered to; no vulnerable groups (e.g. minorities, impaired people, children) were surveyed.

#### 3.3.1 Sample

The sample consisted of 120 German-speaking individuals from Germany. The average participant was 28.60 years (*SD* = 7.70) old, ranging from 18 to 62 years. 49.58 % of all participants identified as female and 49.58 % identified as male, .84 % as diverse. 6.67 % reported they were due to start a new job within the next month. 55.00 % reported to work full-time and 38.33 % reported to work part-time.

#### 3.3.2 Measures

*AI literacy knowledge:* We used the AI literacy test [16] aiming for the assessment of AI literacy (defined as a „basic understanding of AI”) using 29 single-choice items with four options each (only one was correct) and one sorting item. The questions include a wide range of knowledge about the application of AI (e.g., „In which of these areas is AI typically used?“), the capabilities of AI (e.g., „What can weak AI NOT do?“), ethical and legal considerations (e.g., „What legal challenges do AI applications entail?”), and more topics. For the only item that was not single-choice, participants were asked to use drag and drop to sort the process steps during supervised learning in the correct order. All items were recoded (right = 1, wrong = 0). Then, as described by Hornberger et al. [16], we fitted a unidimensional 3-PL item response theory model to the test data using a fixed guessing rate of .25 with the packages *mirt* (Chalmers, 2012). The model showed a good model fit (M2(405) = 408.81, p = .438, RMSEA = .009 (95%-CI: 0, .034), SRMR = .085, TLI = .99, CFI = .99). We used the theta estimates (i.e., estimates for the individual ability) for each participant for hypothesis testing.

*Prior use of AI and Learning about AI*: Prior use of AI was measured using 8 items. Participants were asked if they use different types of AI (e.g., „I use applications in which AI is integrated (e.g. search engines product recommendations.“, „I use AI to revise creative content (e.g. images, audio, videos)", or „I create my own AI applications and work with them.“). Learning about AI was measured with 5 items such as „I inform myself about AI when I find information by chance (e.g. texts, podcasts, videos).” or „I read specialist literature on AI.“. Participants rated each statement using an 11-point Likert scale (0 = “never”, 5 = „several times each month”, 10 = “several times each day”). Both scales showed a good internal consistency (α_Use_ = .86, α_Learning_ = .84).

*AI literacy*: As before, the *Meta AI Literacy Scale* as described in section 2.1 was used to measure AI literacy, this time in a German version. In this sample, all scales of the MAILS showed good or very good internal consistencies (α < .8) except emotion regulation which Cronbach's alpha was still acceptable (α = .77).

#### 3.3.3 Results

We computed Pearson correlations of all MAILS scales with the test scores of the AI literacy test and the scores for prior use of AI and learning about AI (Table 4).

**Table 4:** Pearson correlations between all scales of the MAILS and the tests scores of the AI literacy test and the scores for prior use of AI and learning about AI.

	AI test (theta)	Prior use of AI	Learning about AI
AIL	.21∗	.49∗∗∗	.42∗∗∗
Create AI	.02	.37∗∗∗	.40∗∗∗
AISC	.24∗∗	.09	.10
AISE	.13	.39∗∗∗	.39∗∗∗

Use AI	.17	.50∗∗∗	.36∗∗∗
Know & understand AI	.25∗∗	.50∗∗∗	.43∗∗∗
AI Ethics	.13	.23∗	.29∗∗
Detect AI	.12	.25∗∗	.24∗∗
Emotion regulation	.41∗∗∗	.11	.07
Persuasion literacy	.05	.06	.10
Problem-solving	.12	.34∗∗∗	.30∗∗∗
Learning	.13	.39∗∗∗	.43∗∗∗

Note. ∗*p* < .05, ∗∗*p* < .01, ∗∗∗*p* < .001; AIL = AI literacy, AISC = AI self-competency, AISE = AI self-efficacy.

Only the total AI literacy, AI Self-Competency, Know & understand AI, and emotion regulation were positively related to the test scores. Most correlations were of small effect size, only the correlation of emotion regulation and the test scores were of medium size. All scales of the MAILS except AI Self-Competency and its subscales (emotion regulation and persuasion literacy) were significantly related to the prior use of AI and learning about AI.

#### 3.3.4 Discussion

Confirming axiom 1d, MAILS's total AI literacy score and the AI literacy test were correlated. The MAILS total measure of AI literacy, thus, overlaps with objective knowledge as measured by an AI literacy test. This seems to be due to the correlation of the AI literacy test and the factor Know & understand AI which contains a self-assessment of knowledge about AI. This correlation supports the validity of the AI literacy test, especially concerning its ability to measure a knowledge component of AI literacy. Unlike expected, Emotion regulation and the corresponding superordinate latent factor of AI Self-competency were also correlated with the AI literacy test scores. Presumably, this might be due to the circumstance that emotion regulation is considered to be important for knowledge acquisition independently of the topic of AI [35].

Confirming axioms 2a and 2b, we found positive relationships between AI literacy and the prior use of AI and learning about AI respectively. Again, the validity of the MAILS is supported as the instrument is capable of detecting differences between individuals who use AI more often compared to those who use it less often and between those who learn about AI more often compared to those who learn about it less often. However, these relationships were not found for AI Self-competency and the subscales of emotion regulation and persuasion literacy. The axioms regarding this specific component of the MAILS thus need to be rejected.

### 3.4 Study IV: Development of a short version of the Meta AI literacy scale

The last study's goal was to develop a shortened version of the Meta AI Literacy Scale, which allows efficient assessment of AI literacy. As no standardized procedure for the development of a short version is described in the scientific literature, we rely on the well-established approach to select the best item/items of each sub-factor of the questionnaire. The resulting new measurement procedure must then be validated again using an independent sample level.

Several data sets were combined to find the items most suitable for a short version: data from Study I, Study II, (first measurement point only), and three additional unpublished data sets collected at the end of the winter term 2022/2023, the beginning of the summer term 2023, and the beginning of the winter term 2023/2024 in different university courses respectively. To detect the „best” items, we used the corrected item-scale correlation, loadings on the respective sub-factors in robust maximum-likelihood confirmatory factor analysis (ML-CFA) with lavaan [36], and loadings on the superordinate factors in robust ML-CFA (Appendix A). It is unusual to specify the number of items in a short version in advance. We nonetheless decided to take this approach for one main reason: We wanted to develop an instrument that is as concise as possible, but that still represents all subscales and scales of the MAILS to ensure that it is still true to the underlying theoretical framework of AI literacy. Each level one scale should, thus, be represented by one item and each level two scale by two. Since the MAILS consists of 9 scales at the lower level, we needed at least 9 items. However, as Create AI is also a scale at level two, we need a second item for Create AI. Thus, the short version of the MAILS should consist of 10 items.

Ethical approval of this study was not requested, as risks and harm to the participants are not to be expected in simple surveys on non-sensitive topics. Basic ethical principles were adhered to; no vulnerable groups (e.g. minorities, impaired people, children) were surveyed.

#### 3.4.1 Sample

The sample consisted of *N* = 653 participants. *n* = 134 participants were students from a German University who studied computer sciences or a related subject and took at least one course related to the topic of AI. *n* = 300 German-speaking (Study I) and *n* = 219 English-speaking participants (full data set from Koch et al., sub. of which a part is used in Study II) were contacted via prolific. The average age was 33.60 years (*SD* = 12.86) in the total sample ranging from 18 to 79 years. Most participants were male (50.84 %) or female (47.01). Some participants were diverse (1.23 %) or did not share this information (.92 %).

#### 3.4.2 Measures

*AI Literacy:* All participants answered the MAILS online using the same instructions and 11-point Likert scales. As described for Study I (section 3.1.2), these participants also answered 34 additional items that were generated during the initial development phase and later excluded during the scale refinement. As described for Study II, these participants also answered other AI literacy questionnaire items (items of the following instruments: [11,14,15]) mixed with the items of the MAILS (section 3.2.2). The student samples only answered the MAILS in its published format.

#### 3.4.3 Results

To achieve the goal of a short version with 10 items (described in section 3.4), the items from the current version of the MAILS that best represent their respective factors were selected. We selected one item per sub-factor for the factors AI Literacy, AI Self-Efficacy, and AI Self-Competency and two items for Create AI. Across all analyses (described in section 3.4), a similar set of items was identified. In all models, items 10, 13, 20, 28, 30, and 33 were selected. The other items differed between the models, however, there was still partial overlap (Appendix A).

To compare the different combinations of items, a confirmatory factor analysis was calculated for each item selection using robust maximum-likelihood estimations (Table 5). The first model, with items that were selected based on the corrected item scale correlations, the model fit was good (χ^2^(29) = 171.392, *p* < .001, RMSEA = .092 (90%-CI: .079, .106), SRMR = .041, TLI = .931, CFI = .965). The second (χ^2^(29) = 163.349, *p* < .001, RMSEA = .093 (90%-CI: .079, .107), SRMR = .040, TLI = .930, CFI = .955) and third model (χ^2^(29) = 154.750, *p* < .001, RMSEA = .090 (90%-CI: .076, .104), SRMR = .038, TLI = .936, CFI = .958) also showed good fits. We decided to use the items from the third model (selected based on the CFA with items directly loading on the superordinate scales) as it showed the best RMSEA and SRMR.

**Table 5:** Loadings for a CFA for each item selection based on the prior analyses.

		Loadings for items selected with		
		Corrected item scale correlation	CFA with subscales	CFA without subscales
AIL	AIL03			.80
	AIL04	.78	.77	
	AIL10	.81	.83	.82
	AIL13	.74	.73	.74
	AIL16		.76	.76
	AIL17	.71		
Create AI	AIL19		.90	.90
	AIL20	.93	.88	.88
	AIL21	.89		
AI Self-Efficacy	AIL23	.81		
	AIL25		.82	.82
	AIL28	.80	.84	.84
AI Self-Competency	AIL30	.59	.60	.61
	AIL33	.71	.71	.69

#### 3.4.4 Discussion

The goal of the last study was to develop a short version of the MAILS by selecting the items that represent their respective scales best. Three different approaches were used each of which yielded a selection of 10 items that showed good fit indices in confirmatory factor analyses. In line with the present paper's steps of validation (Study I to III), the validity of the short version should be further analyzed. Next, the efficient use of the short version of the MAILs should be confirmed in practical use cases.

## 4 General discussion

The first goal of this paper was to validate the Meta AI literacy scale as an instrument to measure interindividual and intraindividual differences regarding AI literacy. For this purpose, a total of eight axioms were derived from the literature. The first five axioms referred to the general assumption that the measures from the MAILS are indicators for the individual manifestation of AI literacy and related psychological constructs. The second set of axioms referred to the assumption that the measures from the MAILS can be used to evaluate interindividual differences in AI literacy and related psychological competencies.

### 4.1 Overall assessment of the validation efforts

Research concerning evidence for the validity of the MAILS so far was scarce. The present paper addresses this research desiderata. Beginning with axiom 1a (factorial structure), confirmatory analyses of two independent samples had confirmed the factorial structure of the MAILS already ([10]; Koch et al., sub.). The current study further confirms the axiom 1b regarding the relation of AI literacy as measured with the MAILS and other self-report measures of AI literacy. Prior analyses supported axiom 1c as correlations of the MAILS with attitude towards AI and openness to technology were found [10]. The prior findings regarding negative attitudes toward AI were mixed [10]. In the current study, we were able to further confirm this axiom in Study II. Unlike expected, we could not confirm the axiom in its completeness as we found no relationship between AI Self-competency and a positive attitude towards AI but instead found a correlation between AI self-efficacy with a negative attitude towards AI. We carefully interpret these findings rather as a call for more empirical research on the exact relationship between AI literacy and attitudes towards than consider them a counterargument for the validity of the MAILS. We also were able to confirm axiom 1d regarding the relation of AI literacy as measured with the MAILS and an AI literacy test. As expected, the factor of the MAILS that captures knowledge about and understanding of AI was related to the AI literacy test. More surprisingly, emotion regulation was also related to the test results. However, we do not assume this to be an argument against the validity of the scale. Instead, we expect that emotion regulation facilitates knowledge acquisition independently of the topic of AI [35] explaining this correlation separately from AI self-competency. The current studies were also able to further our understanding of axioms 2a (correlation with the prior use of AI) and 2b (correlation with learning about AI). The MAILS seems capable of detecting differences between individuals that differ regarding these factorfactors. Interestingly, all scales of the MAILS were significantly correlated with the prior use of AI in Study I except emotion regulation which was not related to the use of AI at work. In Study III, however, AI self-competency and its sum scales were not related to the prior use of and learning about AI. In total, given the present and prior evidence, we assume that the MAILS is a valid measurement instrument that can be used to assess AI literacy and measure interindividual and intraindividual differences. Contrary to expectations, the AI self-competence scale showed deviating correlation patterns, which indicates that the skills assessed in this scale should be viewed separately from the rest of the AI competence.

### 4.2 Remarks on the development of the short version

The second goal of this paper was to develop a short version of the MAILS. We used three different approaches to select the item from each subscale that represents their scales best. All three models showed a good model fit in confirmatory factor analyses. We want to encourage the use of the long version of the MAILS where possible as it allows for the in-depth analysis of AI literacy and related psychological competencies including the inspection of subscales. However, the short version of the MAILS can be used by researchers and practitioners in settings where AI literacy is not the central variable of interest and/or resources are limited.

### 4.3 Limitations and future work

Some limitations were already discussed in the discussion sections of Studies I and II (e.g. section 3.1.4, 3.2.4). Furthermore, for the purpose of scale validation, we combined evidence from English and German-speaking samples from different populations (e.g. Germany, United Kingdom). However, there might be differences between both versions that affect the MAILS values and their relationship with other constructs. We did not use structured translation methods (e.g. a), all items were translated jointly by two researchers. More work is needed to ensure the quality of the translated versions and show the equivalence of both the English and the German versions of this AI literacy scale. As one part of the validation process, we used the AI literacy [16] to test if the MAILS can assess a knowledge component of AI literacy. Knowledge, however, is only one limited part of AI literacy which also includes different abilities and competencies. For these abilities and competencies, so far no objective test exists. Future work should aim at developing an AI literacy test that assesses AI literacy in its entirety.

Validation of a measurement instrument is an ongoing process, and more work needs to be done to validate the MAILS. Axioms 1d (differentiation from other related constructs) and 2c (pre-post-differences when learning about AI) still need to be investigated. Similarly, but on a larger scale, the practical benefits and predictive validity of MAILS have yet to be demonstrated. Further axioms targeted at the extrapolation of the MAILS (eg. „Individuals with high test scores are better suited to interacting with artificial intelligence than individuals with a low test score.“) and decision-making using the MAILS (e.g. „Individuals with higher scores are more suited to professions that require interaction with AI.”) need to be explicated and tested. So far, we know that the MAILS measures AI literacy and can detect interindividual and intraindividual differences, but not yet whether these have practical relevance. Lastly, the short version needs to undergo an identical validation process.

## 5 Conclusion

The validation process is central when a new psychological measurement instrument is developed and is to be used in different contexts. Today, however, validity is seen to be connected to a specific interpretation of a measurement instrument's scores and not a characteristic of the measurement instrument itself. Validity is the most important quality criterion needed for trusting a measurement instrument to make important decisions or carry out research projects. Similarly, a short version is important for practitioners and researchers with limited resources. Following the current paper, we can draw positive conclusions regarding these goals as it was possible to confirm most axioms and find a suitable selection of items for a short version. In sum, this publication contributes to the measurement of AI literacy by providing multifaceted evidence for the validity of the MAILS and highlights the need to continue the validation process in future work.

## Declaration of generative AI in scientific writing

No generative AI was used writing this text.

## CRediT authorship contribution statement

**Martin J. Koch:** Writing – review & editing, Writing – original draft, Project administration, Methodology, Investigation, Formal analysis, Data curation, Conceptualization. **Astrid Carolus:** Writing – review & editing, Supervision, Investigation, Funding acquisition, Conceptualization. **Carolin Wienrich:** Supervision, Investigation, Funding acquisition, Conceptualization. **Marc E. Latoschik:** Supervision, Investigation, Funding acquisition, Conceptualization.

## Declaration of competing interest

The authors declare that they have no known competing financial interests or personal relationships that could have appeared to influence the work reported in this paper.

## Figures and Tables

**Table:** 

	Variable	Corrected item scale correlation	Loadings for CFA with subscales		Loadings for CFA without subscales
Use	AIL01	.80	.81	AIL	.80
	AIL02	.87	.87		.82
	AIL03	.87	.89		**.85**
	AIL04	**.89**	**.89**		.84
	AIL05	.86	.86		.83
	AIL06	.83	.84		.80
Know	AIL07	.78	.78		.74
	AIL08	.73	.73		.69
	AIL09	.82	.84		.80
	AIL10	**.82**	**.84**		**.81**
	AIL11	.73	.72		.69
	AIL12	.74	.72		.70
Detect	AIL13	**.81**	**.87**		**.72**
	AIL14	.77	.82		.71
	AIL15	.58	.57		.44
Ethics	AIL16	.73	**.79**		**.74**
	AIL17	**.73**	.76		.69
	AIL18	.71	.73		.66
Create	AIL19	.87	**.87**	Create	**.87**
	AIL20	**.91**	**.92**		**.92**
	AIL21	**.88**	.89		.89
	AIL22	.83	.85		.85
PS	AIL23	**.80**	.82	AISE	.80
	AIL24	.78	.81		.80
	AIL25	.80	**.83**		**.82**
Learn	AIL26	.84	.86		.82
	AIL27	.73	.77		.77
	AIL28	**.87**	**.89**		**.85**
PL	AIL29	.58	.53	AISC	.36
	AIL30	**.75**	**.80**		**.64**
	AIL31	.55	.67		.62
ER	AIL32	.70	.74		.71
	AIL33	**.74**	**.81**		**.76**
	AIL34	.62	.62		.58

Note. AIL = AI literacy, Create = Create AI, AISC = AI self-competency, AISE = AI self-efficacy, Use = Use AI, Know = Know & understand AI, Ethics = AI Ethics, Detect = Detect AI, ER = Emotion regulation, PL = Persuasion literacy, PS = Problem solving, Learn = Learning.

## Appendix A

Corrected item scale correlations and loadings for two CFA (one with and one without subscales) for each item. The highest value per subscale is printed bold.
